# Glutamate reduces glucose utilization while concomitantly enhancing AQP9 and MCT2 expression in cultured rat hippocampal neurons

**DOI:** 10.3389/fnins.2014.00246

**Published:** 2014-08-12

**Authors:** Fabio Tescarollo, Luciene Covolan, Luc Pellerin

**Affiliations:** ^1^Departamento de Fisiologia, Universidade Federal de São PauloSão Paulo, Brazil; ^2^Laboratory of Neuroenergetics, Department of Physiology, University of LausanneLausanne, Switzerland

**Keywords:** brain energy metabolism, mitochondria, lactate dehydrogenase, monocarboxylate transporter, aquaglyceroporin

## Abstract

The excitatory neurotransmitter glutamate has been reported to have a major impact on brain energy metabolism. Using primary cultures of rat hippocampal neurons, we observed that glutamate reduces glucose utilization in this cell type, suggesting alteration in mitochondrial oxidative metabolism. The aquaglyceroporin AQP9 and the monocarboxylate transporter MCT2, two transporters for oxidative energy substrates, appear to be present in mitochondria of these neurons. Moreover, they not only co-localize but they interact with each other as they were found to co-immunoprecipitate from hippocampal neuron homogenates. Exposure of cultured hippocampal neurons to glutamate 100 μM for 1 h led to enhanced expression of both AQP9 and MCT2 at the protein level without any significant change at the mRNA level. In parallel, a similar increase in the protein expression of LDHA was evidenced without an effect on the mRNA level. These data suggest that glutamate exerts an influence on neuronal energy metabolism likely through a regulation of the expression of some key mitochondrial proteins.

## Introduction

The human brain represents only 2% of the total body weight but it accounts for a much larger proportion of the total energy expenditures of the organism (Magistretti, 1999). Under physiological conditions, glucose is considered the major energy substrate taken up and used by the adult brain to supply its energy needs. A linear correlation between glucose utilization and brain activity has been reported for various brain regions in different conditions (Sokoloff, 1993). This tight relationship has formed the basis for the development of some functional brain imaging techniques (e.g., FDG-PET) to visualize changes in brain activity during cognitive tasks. Despite these important developments, the specific fate of glucose at the cellular and molecular levels remains elusive.

Glutamatergic synapses are by far the predominant type of synapses in the central nervous system and constitute the main source of excitatory neurotransmission. Glutamate enhances both glucose transport (Loaiza et al., 2003) as well as uptake and utilization (Pellerin and Magistretti, 1994, 1996a) in cultured astrocytes. As a consequence, a major part of glucose uptake taking place in an activated brain region and visualized by functional brain imaging is due to astrocytic utilization (Pellerin and Magistretti, 1996b, 2012; Figley and Stroman, 2011). Such a view is reinforced by recent observations made *ex vivo* and *in vivo*. First, it was demonstrated in cerebellar slices using fluorescent glucose analogs that both glucose transport and metabolism occur preferentially in Bergmann glia over Purkinje cells while the same observation was made for astrocytes over neurons in hippocampal slices (Barros et al., 2009; Jakoby et al., 2014). In addition, it was shown in experiments performed in anesthetized rats that whisker stimulation caused a significant increase of glucose uptake in astrocytes, but not in neurons, in the activated somatosensory cortex (Chuquet et al., 2010). While these data indicate that a prominent part of glucose utilization take place in astrocytes, they also suggest that active neurons must obtain an alternative energy substrate to cover their enhanced energy needs.

In addition to glucose, neurons are able to use a few alternative oxidative energy substrates that include monocarboxylates such as lactate or the ketone bodies (Pellerin, 2010). The use of these energy substrates by brain cells requires the presence of specific transporters (Pierre and Pellerin, 2005). Indeed, neurons predominantly express the high affinity monocarboxylate transporter MCT2 (Bergersen et al., 2001; Pierre et al., 2002). In addition, at least some populations of neurons also express the aquaglyceroporin AQP9 (Badaut, 2010), a carrier known to allow the passage not only of glycerol but also of lactate (Badaut et al., 2007). So far, few informations exist about the relative subcellular distribution of these two transporters in neurons as well as the possible regulation of their expression in relation with glutamatergic neurotransmission. In this study, we aimed to determine (1) whether glutamate modulates glucose utilization in rat hippocampal neurons, (2) if AQP9 and MCT2 can be co-expressed by neuronal mitochondria, and (3) if glutamate could alter the expression of AQP9 and/or MCT2 in the same neuronal population.

## Materials and methods

### Hippocampal neuronal cultures

All animal experiments were performed in accordance with the Swiss animal welfare laws under the authorization n° VD 1251.4 delivered by the Service de la consommation et des affaires vétérinaires du Canton de Vaud, Switzerland. Hippocampi were carefully dissected from E18 Wistar rat embryos and then the cells were placed in 0.25% Tripsin-EDTA buffer supplemented with 100 mM of DNAse I. In order to stop the effect of trypsin, neurobasal culture medium with 1% fetal bovine serum was added and then cells were mechanically dissociated by gentle passage through fire-polished glass pipets. After centrifugation (5 min, 300 g), pellets of dissociated cells were resuspended in neurobasal culture medium supplemented with B27 (2%), L-glutamine (0.5 mM) and an antibiotic cocktail (Neugene, 0.5%). Viable cells were counted using a Scepter Handheld Automatic Cell Counter (Millipore, Billerica, USA). Cells were plated at a density of 1.5 × 10^5^ cells/well on Poly-D-Lysine coated coverslips in 24-well dishes containing 0.5 mL of culture medium for immunofluorescence experiments and 7.5 × 10^5^ cells/well in Poly-D-Lysine coated 6-well dishes containing 2 ml of culture medium for the other assays. Cells were maintained at 37°C in a 5% CO_2_ atmosphere, without any further changes.

### Cell culture stimulation procedures

After 6 days *in vitro* (DIV), cultured neurons were stimulated by treatment with 100 μM of L-glutamic acid for 1 h (Bliss et al., 2004; Hartz et al., 2009). This treatment was used to mimic the increased neuronal excitability that occurs during an ictal epileptic episode (Churn et al., 1991; Blair et al., 2008; Hartz et al., 2009).

### [^3^H]2DG uptake and lactate release measurement

Primary cultures of rat hippocampal neurons were used at confluence, usually between 6 and 7 days after seeding. 2-Deoxy-d-[1,2-^3^H]glucose ([^3^H]2DG) uptake was determined as described previously (Pellerin and Magistretti, 1994). On the day of the experiment, the culture medium was replaced by serum-free DMEM supplemented with 5 mM glucose, 44 mM NaHCO_3_, 0.06 g/L penicillin, 0.1 g/L streptomycin, and 0.045 mM phenol red (DMEM_5_). Cultured hippocampal neurons were incubated for 2 h at 37°C in a water-saturated atmosphere containing 5% CO_2_/95% air. The medium was then replaced by 2 ml of the same DMEM_5_ medium containing [^3^H]2DG at a concentration of 1 μCi/ml (33 nM). In order to study the effect of stimulation in cultured cells, 100 μM of glutamate were added to the medium 1 h before substitution by the medium containing [^3^H]2DG as well as 100 μM glutamate and the cells were further incubated for 20 min in the same conditions as previously indicated. The reaction was stopped by aspiration of the medium followed by rinsing the cells three times with ice-cold phosphate buffered saline and 0.1 M NaOH/0.1% Triton X-100 was added to lyse the cells. Aliquots of 500 μl were assayed for radioactivity by liquid scintillation counting, while 50 μl aliquots were used for measurement of protein by the method of Bradford (1976). Results, which represent glucose transporter-mediated uptake and subsequent phosphorylation, were calculated by subtracting from total counts the portion that was not inhibited by the glucose transporter inhibitor cytochalasin B (10 μM). The cytochalasin-sensitive uptake accounted for 80% of total uptake. Lactate measurement was performed as previously described (Pellerin and Magistretti, 1994). Briefly, supernatants from 2DG uptake experiments were collected to determine lactate production and release. Two 100 μl aliquots for each supernatant were placed in separate wells of a 96-well plate. To each well was added 100 μl of a solution containing lactate dehydrogenase 70 U/ml (Sigma, Buchs, Switzerland) and NAD 15 mM (Acros organics, Geel, Belgium) in glycine-semicarbazide buffer 0.33 M, pH 10. After 1 hour at 37°C, plates were read at 340 nm to detect production of NADH. A standard curve with known concentrations of lactate was used to determine lactate concentrations, corrected for the amount of protein in the same culture well.

### Mitotracker staining

The red Mitotracker® (Molecular Probes, Eugene, USA), a mitochondrial potential-sensitive dye, was added to the culture medium of cultured hippocampal neurons at 6 DIV at a concentration of 150 nM for 30 min at 37°C. Samples were washed with PBS and fixed in 4% paraformaldehyde for further use in immunolabeling and confocal fluorescence experiments.

### Antibodies

The commercial antibodies used in this work were anti-AQP9 (Santa Cruz Biotechnology, California, USA), anti-lactate dehydrogenase A, (Abcam, Cambridge, UK), and anti-β-tubulin (Sigma, Buchs, Switzerland). The polyclonal anti-MCT2 antibody is a previously characterized homemade antibody (Pierre et al., 2000).

### Immunocytochemistry

Rat hippocampal neurons grown directly on coverslips were used to perform immunofluorescence experiments. After removal of the culture medium, cells were carefully rinsed in phosphate-buffered saline (PBS) at 37°C and directly postfixed in an ice-cold paraformaldehyde fixative (PFA 4% in PBS for 30 min at 20°C). Fixed cells were treated with casein (0.5% in PBS) for 1h at room temperature to block non-specific sites. For immunostaining, cultures were incubated overnight at 4°C in 50μ L of freshly prepared anti-MCT2 antibody solution [anti-MCT2 diluted 1:50 (Pierre et al., 2000) in PBS containing 0.25% bovine serum albumin] and/or with anti-AQP9 diluted 1:5 in PBS containing 0.25% bovine serum albumin. After carefully rinsing in PBS, cultures were incubated for 2 h at room temperature in a solution containing Cy3-conjugated anti-rabbit IgG or FITC-conjugated anti-goat IgG (diluted 1:250; Jackson Immunoresearch, Baltimore, USA). After rinsing in PBS twice and a final rinsing in water, coverslips were mounted with Vectashield containing DAPI (Reactolab SA, Burlingame, CA, USA). Coverslips containing the cells were examined and photographed using a LSM 710 Quasar Confocal Microscope (Zeiss, Hallbergmoos, Germany). Images were obtained using a Plan Apochromat 63× objective and acquired using a cooled CCD camera (Axiocam, Zeiss, Hallbergmoos, Germany), together with the Zeiss 2009 software (Zeiss, Hallbergmoos, Germany). Five neurons were randomly chosen on each coverslip. Pictures were then analyzed using the plugin WCIF-ImageJ for co-localization analysis for ImageJ NIH software (National Institutes of Health Image program, version 1.62, Rockville Pike, USA). Images were converted to 8-bit images for each red and green label of each image, and then the level of co-localization was determined by Mander's overlap coefficient, and the data are presented as percentage of co-localization.

### Electrophoresis and western blotting

Rat hippocampal neurons in each culture dish were homogenized in 500 μL of lysis buffer containing the following: Tris-HCl, pH 6.8, 80 mmol/L; EDTA (ethylene diamine tetraacetic acid), 5 mmol/L; NP-40, 1% or SDS, 5% and a mixture of protease inhibitors (Complete, Roche Molecular Biochemicals, Mannheim, Germany). Control and each stimulated condition were performed in triplicate. Protein samples from rat hippocampal neuronal cultures were sonicated and heated at 95°C for 5 min in half the final volume of SDS-PAGE sample buffer (Tris-HCl, 62.5 mmol/L; DTT, 50 mmol/L; SDS, 2%; glycerol, 10%; and bromophenol blue, 0.1%). Samples were loaded onto polyacrylamide gels composed of a 4.5% acrylamide-bisacrylamide stacking gel and a 12% acrylamide-bisacrylamide running gel. After electrophoresis, proteins were transferred onto nitrocellulose membranes (Trans-Blot Transfer Medium 162-0115; Bio-Rad, Reinach, Switzerland) using a Transblot semi-dry transfer cell (Bio-Rad, Reinach, Switzerland). For protein detection, membranes were incubated in a blocking solution of Tris-buffered saline supplemented with Tween-20 (TBST; Tris-HCl, pH 7.5, 50 mmol/L; NaCl, 150 mmol/L; and Tween-20, 0.1%) containing 5% non-fat milk for 1 h at room temperature. Membranes were incubated overnight at 4°C in a solution containing the primary antibody. After washing in TBST, membranes were incubated with horseradish peroxidase (HRP) conjugated secondary antibodies in TBST containing 1% of non-fat milk, for 2 h at room temperature. After washing in TBST, membranes were submitted to a chemiluminescent reaction using ECL (GE Healthcare, Ecublens, Switzerland), and then photographed using the ChemiDoc XRS + (BioRad). Quantification was made using ImageJ software (NIH, Maryland, USA).

### Immunoprecipitation

Primary cultures of rat hippocampal neurons were collected and homogenized in ice-cold lysis buffer containing 20 mM Tris HCl; 137 mM NaCl; 10% Glycerol; 1% n-octyl-b-D-glucoside (OG) and 2 mM EDTA, pH 7.4, in presence of a complete set of protease inhibitors (Complete; Roche Molecular Biochemicals, Mannheim, Germany) and the homogenate was sonicated three times for 5 s each. The homogenate was reacted with the primary antibody (1:50 anti-MCT2) overnight at 4°C. Agarose-conjugated protein A was added and incubated with gentle rocking at 4°C for 2 h. This complex was then centrifuged at 14,000 g during 30 s at 4°C and washed five times with ice-cold lysis buffer. The pellet was then resuspended in 3× SDS sample buffer and microcentrifuged for 30 s. The sample was heated to 95–100°C for 5 min and centrifuged for 1 min at 14,000 g. The supernatant was collected, loaded on SDS-PAGE and Western blot was performed as described above to verify the presence of AQP9 in the MCT2 immunoprecipitate. Total protein extract from primary cultures of rat hippocampal neurons was loaded as control for each experiment.

### Quantitative real-time polymerase chain reaction

Quantitative determination of AQP9, MCT2, and LDHA mRNA expression levels was performed by quantitative Real-Time Polymerase Chain Reaction (qRT-PCR) according to Heid et al. (1996) using a StepOnePlus or ViiA 7 sequence detection system from Applied Biosystems (Rotkreuz, Switzerland). The following sets of oligonucleotides (Microsynth, Balgach, Switzerland) were used: ActinFo, CTTCTTTGCAGCTCCTTCGT; ActinRe, ATATCGTCATCCATGGCGAAC (Embl: X03672); MCT2Fo, CAGCAACAGCGTGATAGAGCTT; MCT2Re, TGGTTGCAGGTTGAATGCTAAT (Embl: NM_017302); AQP9Fo, GATGGACTCATGGCCTTTGCTG; AQP9Re, CAATCATAGGACCCACGACAGG (Embl: NM_022960.2); LDHAFo, TGGCCTCTCCGTGGCAGACT; LDHARe, CCCCCAGACCACCTCAACACAA (Embl: NM_017025.1).

### Statistical analysis

Experimental data are expressed as mean ± standard deviation (SD). They were statistically analyzed with a non-parametric Mann–Whitney test. Statistical significance was set at *p* < 0.05.

## Results

**Figure 1:**
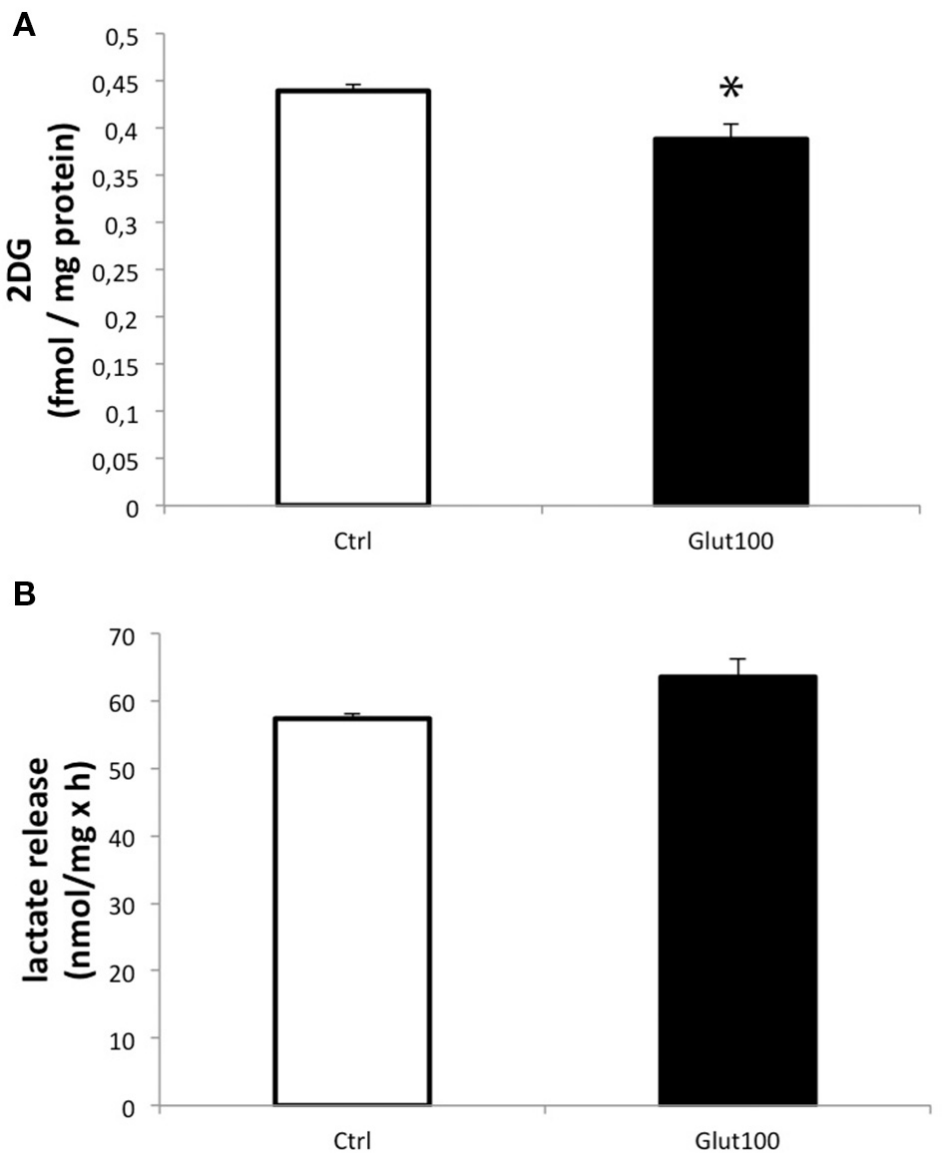
**Effect of glutamate on glucose utilization and lactate production in cultured rat hippocampal neurons**. **(A)** [^3^H]-2-deoxyglucose uptake and accumulation was measured in primary cultures of rat hippocampal neurons for 20 min either without exposure to glutamate (Ctrl) or with prior treatment with glutamate 100 μM for 1 h followed by the presence of the same concentration of glutamate during the [^3^H]2DG uptake measurement (Glut100). **(B)** Lactate production and release was measured in primary cultures of rat hippocampal neurons for 20 min either without exposure to glutamate (Ctrl) or with prior treatment with glutamate 100 μM for 1 h followed by the presence of the same concentration of glutamate during the lactate production measurement (Glut100). Data represent mean ± SD with *n* = 3. Statistical analysis was performed using a non-parametric Mann–Whitney test. An asterisk indicates 2DG uptake significantly different from control with *p* < 0.05. 2DG, 2-Deoxyglucose; CTRL, Control.

Glucose utilization of cultured hippocampal neurons in response to glutamate stimulation was evaluated by measuring ^3H-2-deoxyglucose^ (2DG) uptake. Treatment of cultured hippocampal neurons with glutamate 100 μM for 1 h induced a significant decrease in 2DG uptake (−11.74 ± 3.7%) compared to the control group (Figure 1A). In parallel, measurement of lactate accumulated in the medium did not reveal any significant difference, arguing against a reduction in glycolytic flux ending with lactate production (Figure 1B). Such an unexpected effect of glutamate on glucose utilization in neurons suggested that some modifications, notably on oxidative metabolism, might take place in these cells following an exposure to glutamate. For this reason, the expression of the aquaglyceroporin AQP9 and the monocarboxylate transporter MCT2 which are involved in the transport of oxidative substrates was investigated in this preparation.

**Figure 2:**
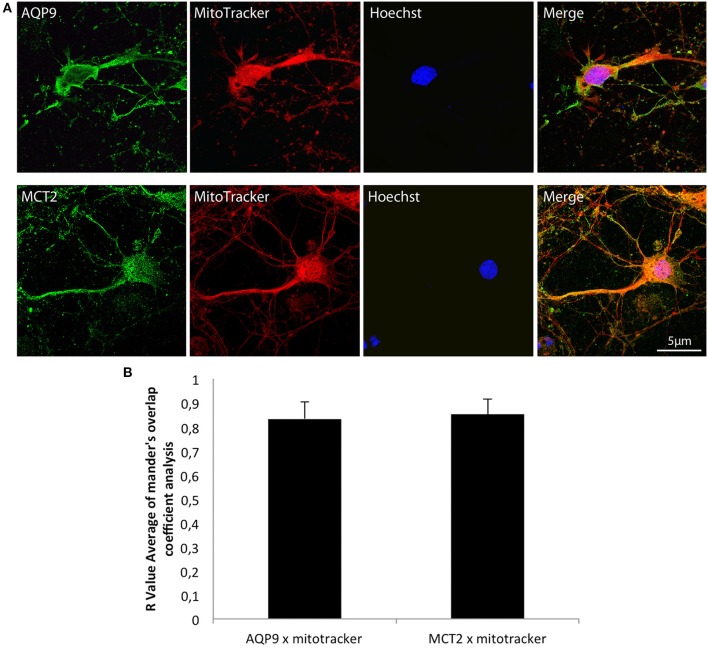
**Immunofluorescent localization of AQP9 and MCT2 in mitochondria of cultured rat hippocampal neurons (A) Immunofluorescent labelings for AQP9 (in green, upper row) or MCT2 (in green, bottom row) and mitotracker staining (in red) in cultured rat hippocampal neurons**. Colocalization between AQP9 or MCT2 and mitotracker appears as yellow on merged images (Merge). Nuclei (in blue) were labeled using Hoechst staining. **(B)** Quantification of the degree of colocalization between AQP9 or MCT2 and mitotracker. The degree of colocalization was assessed with Mander's overlap coefficient obtained by using the plugin WCIF-ImageJ for colocalization analysis for ImageJ NIH software. Immunofluorescence was visualized using confocal microscopy with appropriate filters. Data represent mean ± SD with *n* = 5.

Double labelings with a mitochondrial marker, the MitoTracker®, and either MCT2 or AQP9 were performed on cultured hippocampal neurons and analyzed by confocal microscopy (Figure 2). Observation of fluorescence images revealed that co-localization was present for both AQP9 and MCT2 (Figure 2A). Quantification of the degree of co-localization revealed that it reaches 83.2 ± 7.1% between AQP9 and the mitochondrial marker while it was of 85.2 ± 6.2% for MCT2 and the same mitochondrial marker (Figure 2B).

**Figure 3:**
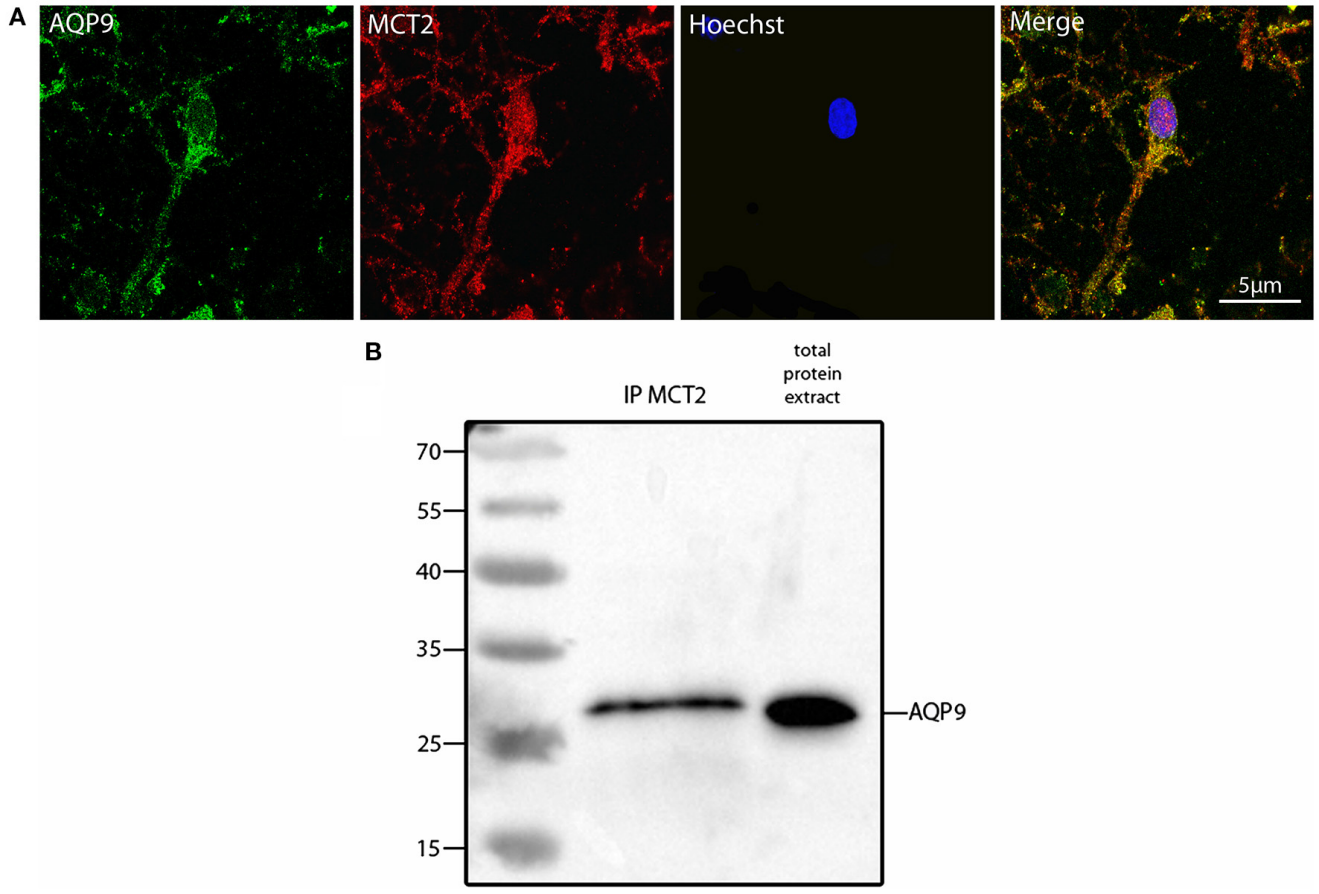
**Colocalization and interaction between AQP9 and MCT2 in cultured rat hippocampal neurons (A) Double immunofluorescence labeling for AQP9 (in green) and MCT2 (in red) performed in cultured hippocampal neurons**. Colocalization between AQP9 and MCT2 appears as yellow on the merged image (Merge). Nuclei (in blue) were labeled using Hoechst staining. Immunofluorescence was visualized using confocal microscopy with appropriate filters. **(B)** Western blot for AQP9 in cultured hippocampal neuron protein extract after immunoprecipitation with anti-MCT2 antibody (IP MCT2) or in total protein extract. This experiment was repeated twice from separate cultures with similar results.

Such a similar degree of co-localization with the same mitochondrial marker suggested that AQP9 and MCT2 could co-localize together. Indeed, using double immunofluorescence labeling and confocal microscopy, it could be observed that AQP9 and MCT2 shared a similar subcellular distribution in cultured hippocampal neurons (Figure 3A). Quantification of the level of co-localization between MCT2 and AQP9 staining in these neurons yielded a value of 83.0 ± 4.5%. Such a high degree of co-localization raised the possibility of a closer interaction between the two proteins. In order to determine whether MCT2 interacts with AQP9 protein, MCT2 was immunoprecipitated from total homogenates of cultured hippocampal cells, protein fractions obtained from MCT2 immunoprecipitates were separated by electrophoresis and analyzed for the presence of AQP9 by western blotting. Results show that AQP9 is indeed detected in the MCT2 immunoprecipitate (Figure 3B), indicating that AQP9 and MCT2 are linked to each other.

**Figure 4:**
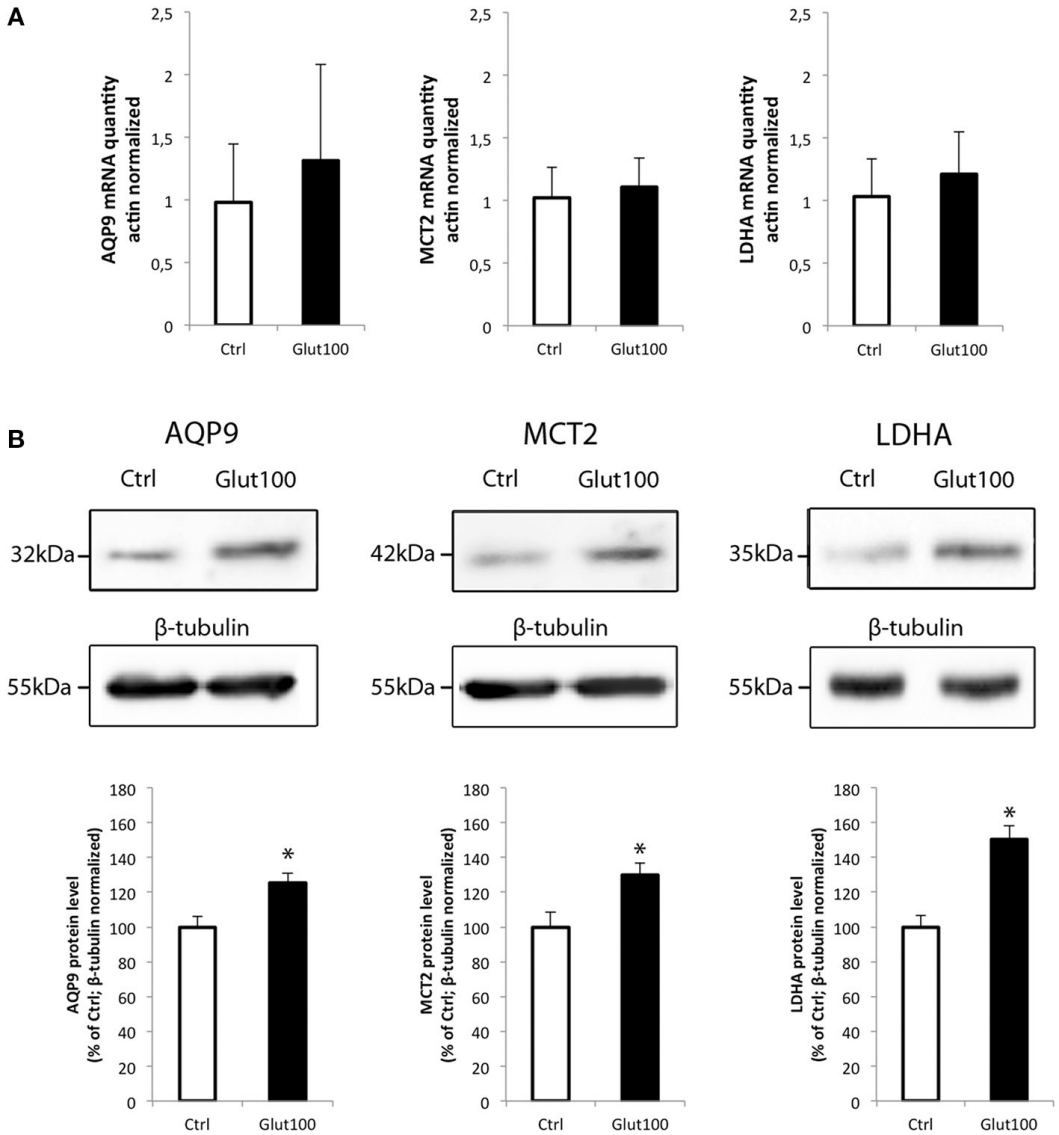
**Effect of glutamate on mRNA and protein expression of AQP9, MCT2, and LDHA in cultured rat hippocampal neurons (A) Primary cultures of rat hippocampal neurons were treated with glutamate 100 μM for 1 h**. Then mRNA was extracted and analyzed for AQP9, MCT2 and LDH mRNA expression using RT-PCR. Results are expressed as relative expression vs. control with mean value for control set at 1 after values had been normalized using β-actin as internal reference. Results represent mean ± SD with *n* = 3. The experiment was repeated three times from different cultures with similar results. Statistical analysis was performed using a non-parametric Mann–Whitney test. No significant difference was observed. **(B)** Primary cultures of rat hippocampal neurons were treated with glutamate 100 μM for 1 h. Then proteins were extracted and analyzed for AQP9, MCT2, and LDH protein expression using Western blot. Blot pictures were obtained with Bio-Rad Chemidoc™ XRS system (BioRad, Cressier, Switzerland) and quantified using ImageJ software. Quantitative results are expressed as percentage of control after the values had been normalized using β-tubulin signal as reference. Results represent mean ± SD with *n* = 3. The experiment was repeated three times from different cultures with similar results. Statistical analysis was performed using a non-parametric Mann–Whitney test. An asterisk indicates protein level significantly different from control with *p* < 0.05.

The possibility that the level of expression of these two energetic substrate transporters could be regulated by glutamate has been investigated in rat hippocampal cultured neurons. In parallel, the expression of the isoform A of lactate dehydrogenase, a key enzyme for the use of lactate as oxidative substrate, was also determined. First, exposure of cultured hippocampal neurons to glutamate (100 μM) for 1 h caused no significant change in the expression level of either AQP9, MCT2, or LDHA (Figure 4A). A similar analysis was performed at the protein level. In this case, it could be observed by Western blot that AQP9, MCT2, and LDHA protein expression was enhanced in cultured hippocampal neurons after exposure for 1 h to glutamate 100 μM (Figure 4B, upper panel). Quantification of the level of expression revealed increases of 25.3 ± 1.46% (AQP9), 29.9 ± 1.98% (MCT2), and 50.33 ± 3.93% (LDHA) when compared to control.

## Discussion

Glutamate is the main excitatory neurotransmitter in the central nervous system. It is purported that its depolarizing action on neurons should lead to increased energy demands in this cell type. Indeed, it was reported that activation of NMDA receptors (a subtype of glutamate receptors) causes an increase of glucose utilization in cerebellar neurons (Bak et al., 2009). In contrast, exposure of rat hippocampal neurons to glutamate induced a decrease in glucose transport (Porras et al., 2004). Our data showing a decrease in glucose utilization in rat hippocampal neurons after exposure to glutamate are consistent with the study of Porras et al. Although the contradictory results between the finding of Bak et al. on one hand, and ours as well as the study of Porras et al. on the other hand could be explained by the use of a different preparation of neurons (cerebellar vs. hippocampal) as well as by different stimulation conditions (brief NMDA applications in Mg-free medium for Bak et al. vs. constant glutamate application at 500 μM for Porras et al. or 100 μM for us), other reasons can be invoked. Thus, it was recently shown that activation of NMDA receptors in rat cortical neurons led to an enhancement of glycolysis at the expense of the pentose phosphate shunt (Rodriguez-Rodriguez et al., 2012). Such an effect, which is due to the stabilization of the enzyme 6-phosphofructo-2-kinase/fructose-2,6-bisphosphatase-3 (PFKFB3), a key regulator of glycolysis, eventually causes oxidative stress, and cell death (Rodriguez-Rodriguez et al., 2013). Based on these results, it can be concluded that specific NMDA receptor activation enhances glucose utilization in neurons by activating glycolysis (with the risk of inducing cell death later on) while glutamate exposure (which can activate several subtypes of glutamate receptors in parallel) rather leads to reduced glucose utilization by an as yet unknown mechanism.

If we consider that glucose utilization and glycolysis could be reduced upon physiological stimulation with glutamate, then neurons must rely on another energy substrate to face the enhanced energy demands caused by glutamate-dependent depolarization. One possibility could be to use monocarboxylates such as lactate, pyruvate, or the ketone bodies β-hydroxybutyrate and acetoacetate. Indeed, it was previously shown that neurons preferentially use lactate over glucose-derived pyruvate as preferential oxidative energy substrate, both *in vitro* (Bouzier-Sore et al., 2003, 2006) and *in vivo* (Tyson et al., 2003; Serres et al., 2004, 2005; Gallagher et al., 2009; Boumezbeur et al., 2010; Wyss et al., 2011). In order to favor monocarboxylate utilization, specific transporters must be present. It was previously shown that MCT2 is the predominant neuronal monocarboxylate transporter (Bergersen et al., 2001; Pierre et al., 2002). MCT2 was found to be particularly enriched in dendrites, not only at the plasma membrane associated with the postsynaptic density but also in an intracellular pool (Pierre et al., 2009). Moreover, its subcellular distribution was shown to be altered by various stimuli including glutamate, leading to enhanced lactate uptake (Pierre et al., 2009). In parallel, the presence of the aquaglyceroporin AQP9, which can transport small oxidative subtrates such as lactate or glycerol, has been reported in neurons. Thus, its expression was described in some populations of neurons such as catecholaminergic (Badaut et al., 2004) as well as hippocampal and cortical neurons (Mylonakou et al., 2009; Arciénega et al., 2010). The presence of both MCT2 and AQP9 in neuronal mitochondria has been documented. In rat brain sections, MCT2 was found to co-localize with cytochrome oxidase in the inner membrane of neuronal mitochondria in the cortex, the hippocampus and the thalamus (Hashimoto et al., 2008). Similarly, AQP9 was found to be present in brain mitochondrial inner membranes (Amiry-Moghaddam et al., 2005) although the presence of AQP9 in mitochondria has been disputed (Yang et al., 2006). Our results not only confirm that both MCT2 and AQP9 are present in rat hippocampal neuron mitochondria, but they also co-localize and co-immunoprecipitate. These results suggest that both transporters are probably part of a mitochondrial complex that facilitates the entry and use of oxidative substrates. Our observation that glutamate enhances the expression of both MCT2 and AQP9 expression would be consistent with idea that to face enhanced energy demands, neurons would favor the use of extracellular monocarboxylates as oxidative substrates in mitochondria at the expense of glucose. In our case, it could be pyruvate which is present in the culture medium.

Interestingly, the expression of both MCT2 and AQP9 proteins together with the LDHA protein was enhanced after treatment with glutamate, while no change in mRNA expression for all three genes was detected. This observation suggests that a coordinated translational regulation occurs for these three proteins, reinforcing the view that they might form together a complex within mitochondria, as suggested before for MCT2 and LDH (Hashimoto et al., 2008). Previously, it was shown that MCT2 is subject to translational regulation in mouse cortical neurons when treated with different neuroactive substances (Pierre et al., 2003; Chenal and Pellerin, 2007; Chenal et al., 2008; Robinet and Pellerin, 2010). However, it is the first time that both AQP9 and LDHA are shown to undergo a similar regulation in cultured neurons. One possible explanation for the concomitant upregulation of these three mitochondrial proteins by glutamate could be to facilitate the use of oxidative substrates other than glucose (as glucose utilization is decreased under such conditions) in order to face the increased energy demands imposed by excitation. Nevertheless, it remains to be demonstrated whether the oxidation of monocarboxylates and/or glycerol are enhanced under these conditions.

### Conflict of interest statement

The authors declare that the research was conducted in the absence of any commercial or financial relationships that could be construed as a potential conflict of interest.
